# Exploring the Role of Gender in the Public Health Supply Chain Workforce in Low- and Middle-Income Countries

**DOI:** 10.9745/GHSP-D-24-00232

**Published:** 2025-05-09

**Authors:** Susan Truog, Katie Reynolds, Rebecca Alban, Louis Tshituka, Tafwirapo Chihana, Mariam Zameer, Amanda Pain, Bvudzai P. Magadzire, Sierra Petrosky

**Affiliations:** a VillageReach, Seattle, WA, USA.; b VillageReach, Spain.; c VillageReach, Kinshasa, Democratic Republic of Congo.; d VillageReach, Lilongwe, Malawi.; e VillageReach, South Africa.

## Abstract

This article describes exploratory research conducted to understand pathways to entering the public health supply chain (PHSC) workforce in low- and middle-income countries (LMICs) to identify potential barriers for women working in this field and gather suggestions on how to improve gender equity in the PHSC workforce. Key informant interviews were conducted in the Democratic Republic of Congo and Malawi with health sciences students, health science education professionals, individuals currently working in the PHSC workforce, and global stakeholders. An online survey was conducted with responses from PHSC professionals across 26 countries. The survey and interviews revealed that respondents perceived that there were gender imbalances and inequities at all levels of the PHSC workforce. Respondents reported on barriers for women to receive the education, training, mentorship, and leadership opportunities needed to advance in this profession, barriers to traveling for work due to cultural norms and safety concerns, and a lack of policy and structural support for women to feel safe and supported at work. To improve gender equity in the PHSC workforce, we recommend strengthening career pathways for women; fostering mentorship opportunities among women; making recruitment practices more gender sensitive; implementing gender-sensitive policies; improving access to financial, physical, and technological resources; collecting and using gender-disaggregated data; and increasing the number of women in leadership positions.

## Abstract (precis)

We identified potential barriers for women entering the health supply chain workforce and provide recommendations on how to improve gender equity in the health supply chain workforce.

## Teaser

**Key Findings:**

- Gender inequities in the public health supply chain (PHSC) workforce exist in education, training, mentorship, travel, and leadership opportunities.
- Social and cultural norms around appropriate professions for women, domestic responsibilities, and work-related travel were found to hinder women’s entry, retention, and advancement in the PHSC workforce.

**Key Implications:**

- Public and private supply chain employers should improve recruitment and promotion practices to be more gender-sensitive and work with academic institutions to strengthen career pathways for women, including increasing awareness of PHSC professions.
- National stakeholders should implement gender-sensitive policies, including antidiscrimination, parental leave, and sexual harassment policies.

## BACKGROUND

### Understanding the Supply Chain Workforce

The health supply chain workforce comprises all the people who select, procure, store, and distribute medicines, vaccines, and other health products. In Africa, this includes pharmacists, logisticians, supply chain managers, data managers, and warehouse and transport personnel, as well as doctors, nurses, and other clinical and administrative staff who have supply chain responsibilities in addition to their regular functions. The health supply chain workforce is often unrecognized as a distinct profession within health care systems in Africa,^1^ which means both men and women in these roles lack the specialized skills needed to do their work effectively and efficiently, and Ministries of Health often do not adequately plan for or support these roles.^2^

Supply chain professionals work across all levels of the supply chain—from health facilities to national-level Ministry of Health decision-makers who determine supply chain policies, priorities, and budgets.^2^ Managing high-performing supply chains that are equitable, people centered, resilient, and sustainable requires a professional supply chain workforce that is motivated and equipped with the right skills and working conditions.^2^

Gender inequities are prevalent throughout the global health workforce. Women are the backbone of these systems, comprising 67% of the global health and social care workforce^3^ and performing 76% of unpaid work.^4^ A 2024 World Health Organization (WHO) report noted that women have traditionally been excluded from certain medical professions (e.g., physicians), and women’s health work has often been considered a “semi-profession.”^3^ The degree to which health and care work is underrecognized and undervalued is compounded by a lack of gender-disaggregated data on wages, employment conditions, and unpaid work, meaning gender imbalances in the health workforce are inadequately reported and acted upon.^3^ Women in the health workforce today remain underrepresented in leadership positions and overrepresented in unpaid work, earning 27% less than men on average globally.^3^^,^^4^

Gender inequities in the health workforce are especially pronounced in Africa, where women make up only 28% of physicians.^5^ A gender analysis of the health care workforce in Sierra Leone, Uganda, and Zimbabwe demonstrated that women had less access to training due to household responsibilities that limited their travel outside the home, which subsequently limited career advancement opportunities and income growth. ^6^

### Gender and Supply Chain Workforce

Women are underrepresented in supply chain management, making up 43% of the global public health supply chain (PHSC) workforce and only 27% of supply chain management positions.^7^ Given that women and children are the primary beneficiaries of health programs, women’s perspectives are important in ensuring that supply chains are designed with women’s needs and preferences in mind.^4^ For example, during the COVID-19 pandemic, predominantly male decision-makers deprioritized family planning products, resulting in stock-outs that caused an estimated 1.4 million unintended pregnancies worldwide.^8^ There are numerous examples of male-dominated supply chain decision-making in humanitarian emergencies resulting in the exclusion of gender-sensitive products (such as menstrual hygiene products) and misjudging the needs of women, causing wasteful spending and poor quality services for women.^9^

Women make up 43% of the global public health supply chain workforce and 27% of supply chain management positions.

Women often seek services on behalf of their children (e.g., immunization) in addition to their own needs, so it is critical for both male and female supply chain professionals to understand both male and female clients and consider their needs in supply chain planning and decision-making.^10^ The intersection of gender and health supply chains and particularly the role that women logisticians play in shaping health supply chains in low- and middle-income countries (LMICs) is underexplored in current published literature.

### Supply Chain Career Pathways

**FIGURE 1:**
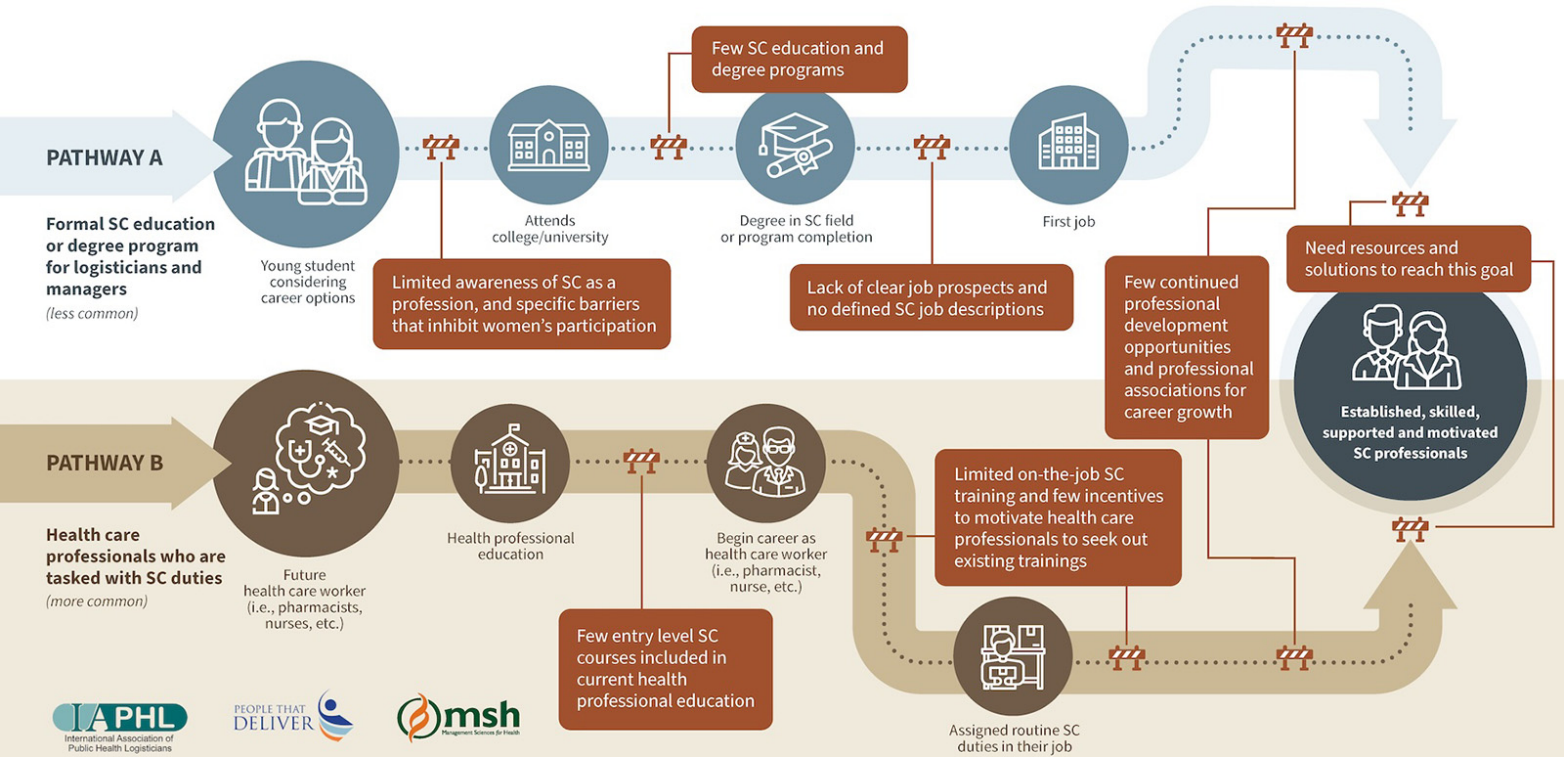
PHSC Career Pathways and Barriers to Building and Advancing a Career Abbreviations: PHSC, public health supply chain; SC, supply chain.

Many African countries do not have specific educational programs for health supply chain logistics.^11^ There are generally 2 health supply chain career pathways (Figure 1). The first pathway to becoming a supply chain professional (Pathway A) is less common, whereby the individual obtains a formal supply chain education. The second and more common pathway to becoming a supply chain professional (Pathway B) is for health care workers, such as pharmacists and nurses, to be assigned supply chain duties as part of their role. As Figure 1 illustrates, the pathway to becoming an established supply chain professional is filled with challenges and barriers, beginning at the education stage and continuing through career entry and career advancement.

To better understand and address gender inequities along these career pathways, VillageReach led exploratory research to (1) describe potential pathways to entering the PHSC workforce in LMICs and identify potential barriers for women, (2) explore differences and similarities in the experiences of men and women who are currently working in PHSC, and (3) gather suggestions on how to improve gender equity in the PHSC workforce.

## METHODS

### Conceptual Framework

**FIGURE 2:**
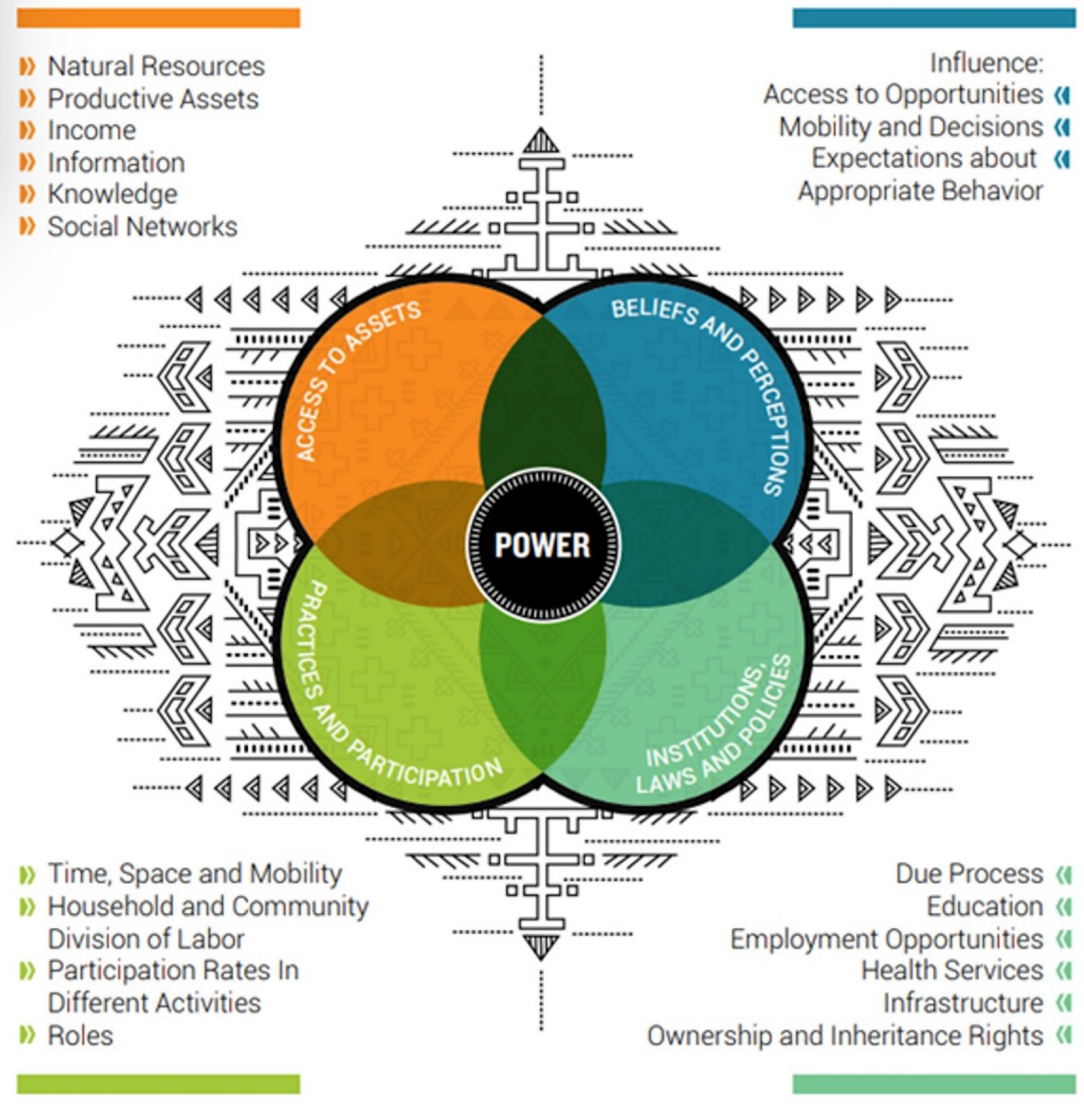
Gender Analysis Framework Used to Identify and Understand Inequities in PHSC Workforce Abbreviation: PHSC, public health supply chain. Source: Jhpiego.^13^

Gender analysis is a powerful approach to identify, understand, and address inequities in health systems.^3^ We assessed several gender analysis frameworks and conceptual models and chose Jhpiego’s Gender Analysis Framework^12^ as we could adapt it to the PHSC workforce to meet our research objectives (Figure 2). The framework consists of 4 domains: access to assets; practices and participation; beliefs and perceptions; and institutions, laws, and policies.

We adapted this framework to meet our research objectives and develop 3 research questions: (1) How does the social and cultural context influence entry and retention of women in the PHSC workforce? (2) What are women’s experiences throughout the educational and career pathways to the PHSC workforce? (3) How does the enabling environment currently affect gender balance in the PHSC workforce?

### Country Selection

To facilitate primary qualitative data collection, we considered the African countries where VillageReach operates. We analyzed gender-related indicators available in the World Bank World Development Indicators DataBank^13^ and the Global Gender Gap Index reported by the World Economic Forum.^14^ We believed that the Democratic Republic of Congo (DRC) and Malawi would likely offer distinct perspectives. In DRC, achievement on key gender-related development indicators is typically lower compared to the average in sub-Saharan Africa, while in Malawi, achievement on these indicators is typically slightly higher (Table 1).

**TABLE 1.** Gender-Related Development Indicators in DRC, Malawi, and sub-Saharan Africa

**Gender Analysis Framework Domain**	**Gender-Related Development Indicators**	**DRC**	**Malawi**	**Sub-Saharan Africa**
Access to assets	Literacy rate, youth (ages 15–24 years), gender parity index (values <1 indicate more male youth are literate, values >1 indicate more female youth are literate)^a^	0.88	1.01	0.94
Practices and participation	Employment to population ratio (older than 15 years, female)^a^	58%	65%	55%
Beliefs and perceptions	Women who believe a husband is justified in beating his wife (and of 5 reasons)^a^	75%	16%	N/A
Institutions, laws, and policies	Proportion of seats held by women in national parliaments^a^	13%	23%	24%
Power	Proportion of women subjected to physical and/or sexual violence in the last 12 months (percentage of ever-partnered women ages 15–49 years)^a^	37%	24%	N/A
Overall	Global Gender Gap (0 to 1, higher is better)^b^	0.58	0.67	0.68

Abbreviations: DRC, Democratic Republic of Congo; N/A, not applicable.

a World Development Indicators.^14^

b Global Gender Gap Report.^15^

We conducted an online survey of PHSC professionals from multiple countries and key informant interviews (KIIs) with respondents in DRC and Malawi and global stakeholders.

### Online Survey

The online survey was designed to understand whether a gender imbalance may exist in PHSC education and the PHSC workforce. The survey was conducted using SurveyMonkey in April 2023 and sent to members of the International Association of Public Health Logisticians. A total of 69 members responded (56 respondents provided their gender; 45% of these were women), representing 18 African countries and 8 additional countries. The highest number of responses from a single country came from Ethiopia (8), followed by Nigeria (7) and Zimbabwe (4). Survey results were analyzed for frequency and provided a benchmark but are not meant to be representative of the global PHSC workforce or the International Association of Public Health Logisticians membership.

### Key Informant Interviews

We conducted 68 KIIs with global stakeholders and in-country stakeholders in DRC and Malawi who had direct experience in the PHSC workforce (Table 2).

**TABLE 2.** Key Informant Interviewees With PHSC Experience

			**Gender**	
**Key Informant**	**Country**	**Affiliation**	Female, No.	Male, No.
Global stakeholder	Global		10	0
Health sciences education			20	4
Students	DRC	ISTM Kinshasa Health Organization Management, Health Logistics track	9	0
	Malawi	MCHS, KUHS, Progressive Care Institute	8	0
Administrators	DRC	ISTM Kinshasa Health Organization	0	3
	Malawi	Ekwendeni College of Nursing and Midwifery, Holy Family College of Nursing, KUHS, MCHS	3	1
PHSC professionals			21	13
	DRC	National MOH	2	3
		Provincial MOH	6	1
		Health facility	4	1
		NGO	0	1
	Malawi			
		National MOH	1	2
		Provincial MOH	7	4
		Health facility	1	1

Abbreviations: DRC, Democratic Republic of Congo; ISTM: l’Institut Superieur Technique Medical; KUHS, Kamuzu University of Health Sciences; MCHS, Malawi College of Health Sciences; MOH, Ministry of Health; PHSC, public health supply chain.

We interviewed 10 female global stakeholders, including representatives of the Africa Resource Centre, Gavi, John Snow, Inc., UNICEF, People That Deliver, and the Gates Foundation. Respondents were asked to describe the challenges women encounter in the PHSC workforce along with existing policies, solutions, or strategies designed to improve gender balance in the PHSC workforce.

A total of 17 female health sciences students whose general interests aligned with a career in PHSC were interviewed. In Malawi, students and educators in pharmacy programs were interviewed from 4 different universities, as pharmacists (degree holders), pharmacy technicians (diploma holders), and pharmacy assistants (certificate holders) perform the majority of supply chain and logistics work. We selected 8 female students. In DRC, students and educators were interviewed from l’Institut Superieur Technique Medical (ISTM), a public institution in Kinshasa that has one of the few supply chain-specific degree programs in Africa. ISTM offers a degree in Management of Health Institutions as a 3-year bachelor’s degree or a 2-year master’s degree. We selected 9 female students from this program. All student respondents were asked about gender balance in their education programs, their experience as female students, and how they chose their educational program.

To learn about the current academic environment and the environment needed to support female students in successfully completing their programs, we interviewed 7 academic advisors, administrators, and professors in health sciences. We selected educators and administrators from the same institutions that female student respondents attended (3 administrators in DRC and 4 in Malawi). Respondents were also asked about social and cultural norms that impact the types of PHSC jobs female students pursue.

We interviewed 34 PHSC professionals about their role, education, and PHSC-specific training. They were also asked about any instances of gender-based discrimination in the workplace and their awareness of protocols to report this in the workplace. We interviewed 18 professionals in DRC and 16 in Malawi.

VillageReach hired and trained consultants to lead data collection and transcription in both countries. The interviewers sampled from across the possible institutions to get a mix of perspectives, using snowball sampling to finalize the set of participants. Interviews were conducted in October 2022 in DRC and in December 2022 and January 2023 in Malawi. All interviews were transcribed, translated, and provided to VillageReach’s global research team for analysis, which developed a list of codes using a deductive approach based on the Jhpiego Gender Analysis Framework. Subsequently, a thematic analysis was conducted by reviewing all of the content by code and pulling out key connections, patterns, and emerging themes. This thematic analysis was then distilled into the key findings of the research.

### Ethical Approval

Ethical approval was granted by the Kinshasa School of Public Health Ethical Review Committee in DRC and the National Health Sciences Research Committee in Malawi.

## RESULTS

**FIGURE 3:**
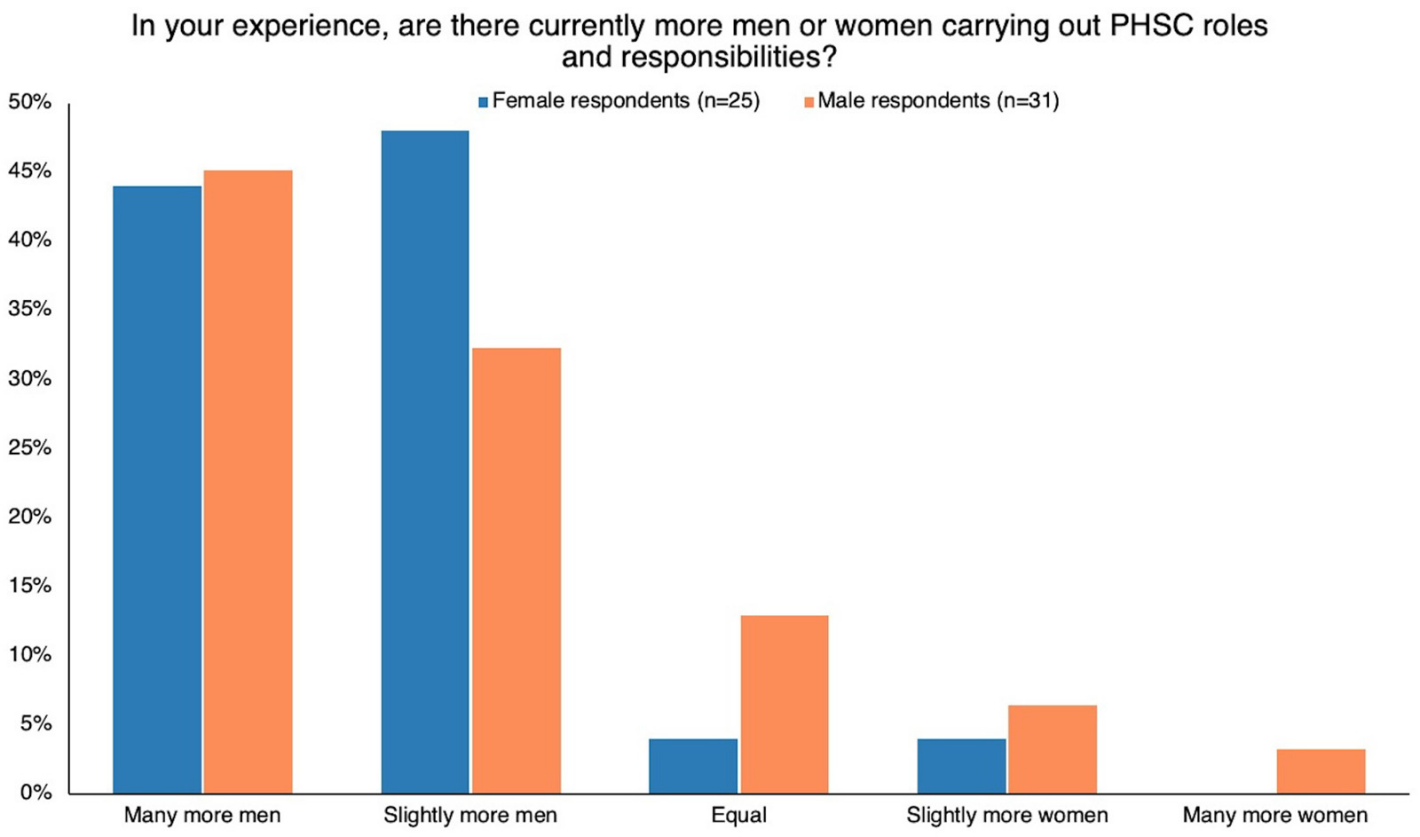
Survey Respondents’ Perceptions About Whether More Men or Women Had PHSC Roles and Responsibilities Abbreviation: PHSC, public health supply chain.

The online survey responses showed that respondents perceived that more men than women were carrying out PHSC roles and responsibilities, with 77% of male respondents and 92% of female respondents reporting that they perceived that slightly or many more men carry out PHSC responsibilities (Figure 3).

When asked how important exploring gender in the PHSC workforce is on a scale of 1 to 10, both male and female respondents agreed that it is a “highly important” issue, with male respondents rating its importance at an average of 7.1 of 10 and female respondents rating its importance at 7.6 of 10.

Survey respondents agreed that exploring gender in the PHSC workforce is a highly important issue.

The KIIs globally and in DRC and Malawi explored the reasons for the perceived gender imbalance. We categorized the findings across the PHSC career pathway: education, careers, and advancement in careers. At each stage, the findings suggest that social and cultural norms, as well as practical and environmental factors, hinder women’s participation in the PHSC workforce.

### Education

#### Awareness of Public Health Supply Chain Profession

Respondents from DRC and Malawi agreed that there was limited awareness and understanding of the supply chain profession. Most current PHSC professionals in both Malawi and DRC reported following the career path of a supply chain “practitioner” (Figure 1, Pathway B), either learning supply chain skills on the job or participating in training after employment. Both male and female students interviewed noted they were not aware that being a pharmacist also included procurement, storage, and tracking of medication. Global PHSC professionals observed a similar lack of awareness of supply chain as a distinct career. However, a professional noted a shift in awareness around the importance of PHSC as a distinct profession because of the COVID-19 pandemic.

#### Gender Norms

Social and cultural norms shape ideas about how women and men should act in their personal and professional lives. They are often internalized early in life and can establish a life cycle of gender socialization and stereotyping.^15^ In both DRC and Malawi, respondents reported having a perception that girls were expected to perform more poorly than boys in math and science courses. This perception affected the types of degree programs women apply for because math and science courses are foundational for entry into pharmaceutical or other health degree programs.

*From lower levels of education, I think [young girls] are not properly guided, they drop subjects because…[they say] a subject is difficult …Pharmaceutical sciences, it involves chemistry, math, physics – so I think women don’t do well in these.* —Male PHSC professional, Malawi

This rhetoric affects girls’ educational and professional choices. A female pharmacy student from Malawi responded that girls often grow up thinking that if they want to work in health care, they can only be nurses.

*I think it’s just fear of [the] unknown. Mostly we females, we look down on ourselves. We also easily believe what others are saying, so when one says that the course is difficult, we plant it in our heads that indeed it is hard.* —Female PHSC professional, Malawi

In addition, a female DRC student respondent said that because women only like to do what is “easy,” they do not explore degree programs where the coursework is seen as advanced or more difficult. These demonstrate the pervasive social and cultural norms around women’s education and, at times, are internalized by women themselves. Respondents noted that trends in enrollment are starting to shift, but further examination may be needed to understand the current enrollment shift in DRC.

#### Structural and Financial Barriers

An important finding was that both male and female students experienced challenges related to having the ability to pay educational fees, finding adequate housing close to campus, and purchasing necessary supplies for school. These barriers were reported for the students participating in this study but are likely to reflect the experience of many tertiary students in LMICs. Though 4 of the Malawi schools had on-campus housing, a student interviewed mentioned that there was not enough on-campus housing. The students had to reapply for housing each year during the 4-year degree program, and returning students were often prioritized for housing, which meant new students were not guaranteed on-campus housing. This meant that students needed to have finances for daily transportation fees in addition to other school expenses. ISTM did not have on-campus student housing, so students were required to find accommodation in the city, as it was not feasible to commute daily to campus from the outskirts of Kinshasa.

While all students face these challenges, they are heightened for women. For example, a male educator in DRC noted that students needed computers, access to the Internet, and mobile phone credits to download course material. Women have less access to these resources. In LMICs, women are 7% less likely than men to own a phone and 16% less likely to have access to mobile Internet.^15^ In DRC, only 16% of women reported having Internet access compared to 35% of men.^16^

Respondents in DRC and Malawi noted that financial assistance through scholarships was limited or nonexistent for both genders. Students obtained scholarships on their own by informally asking community or business leaders for assistance. Educators interviewed noted this led to inequitable access to scholarships because men tended to be more willing to seek financial support informally, and women worried about being exploited when soliciting funding. Overall, respondents reported that although everyone is affected, a lack of access to housing, supplies, and scholarships disproportionately impacts women.

#### Female Role Models

Educators noted that female role models were important for encouraging more women to enroll in PHSC degrees. One educator observed that when female students were sent to rural health centers for their practicum assignment, young girls may increase their awareness of PHSC roles.

Having the support of female role models allows female students to see someone like them performing PHSC roles and responsibilities. These role models can help students navigate challenges and encourage their enrollment in relevant educational pathways, retention once performing PHSC roles, and advancement in the PHSC workforce.

*I was motivated because a certain lady is certified in Supply Chain [by a global professional body]…I even talked to her on Facebook…she told me everything, and if all goes well, this year I will start…the only issue is that women, we look down on ourselves. Like that course, women say that the exams are tough, but this lady encouraged me not to listen to what people say, the only solution is to work hard.* —Female PHSC professional, Malawi

### Careers

#### Hiring Practices

Social and cultural norms and barriers disproportionately affecting women in career preparation contribute to a gender imbalance in the PHSC workforce. Many respondents perceived that PHSC jobs were best suited for men. Because of a lack of awareness of PHSC roles, these roles were believed to mostly involve driving large trucks and lifting heavy boxes of health products, which many perceived as too physically challenging for women. In addition, a gender imbalance in PHSC education would lead to a gender imbalance in hiring. A male PHSC professional from DRC noted that he could only hire based on a candidate’s credentials and that he often did not receive any women candidates with appropriate qualifications. A similar trend was noted in Malawi by a male PHSC professional.

Social and cultural norms and barriers disproportionately affecting women in career preparation contribute to a gender imbalance in the PHSC workforce.

*Pharmacy technicians should [fill the role]. I think we have had only one lady who is a technician who was by quality available for that position.* —Male PHSC professional, Malawi

#### Work-Life Balance

The daily responsibilities of many women as wives and caregivers created a practical challenge of balancing work and household duties with a PHSC career. Female respondents from DRC and Malawi described getting up early or going home to make dinner during their lunch breaks to ensure they completed their household duties. While these challenges for women are not unique to the PHSC profession, the travel expectations for these positions made balancing work and household responsibilities difficult. One male PHSC professional from Malawi noted that, in addition to household responsibilities, women also have responsibilities to their community that prevent them from working.

*In your neighborhood if there is a funeral, they would expect that [a woman] should be there all the time with them. The moment you prioritize your job, I think you are taken as somebody who is not considerate… females would suffer more [than men], because they are the ones expected to be participating more in community activities.* —Male PHSC professional, Malawi

#### Traveling for Work

A female student from DRC said that female PHSC professionals who travel for work were often viewed negatively by communities as some may believe that they were being unfaithful to their husbands. The same community perceived men traveling for work as working hard to support their families. In addition, women were expected to seek their husbands’ permission to travel.

*The law says: a married woman cannot go to work far from her husband’s residence and even if you have the skills, you will be assigned elsewhere.* —Male educator in health logistics program, DRC

To transport supplies and conduct supervision visits, PHSC professionals often travel by motorcycle. While respondents noted that women were capable of driving a motorcycle, many thought women were hesitant to do so due to safety, especially during the rainy season when roads were more difficult to navigate. Travel often requires an overnight stay, and male PHSC professionals were typically able to sleep at rural health clinics; women may not have viewed this option as acceptable, given the social norms around sleeping in unsecured areas and competing household responsibilities.

#### Harassment and Workplace Safety

Female PHSC professionals reported instances of sexual harassment at work, and students and educators agreed this was an issue in the university system. A female PHSC professional in DRC recounted that when a male coworker made romantic advances toward a female coworker, and she was not receptive, the work environment for the woman became hostile. Respondents noted that there were no official sexual harassment policies or clear reporting procedures within the public health care system. One university had an established system, but the process involved female students reporting harassment to a male authority figure. With no recourse available, female PHSC professionals must choose between exiting the work environment or continuing in an environment that does not have protections in place for them.

### Advancement in Careers

Similar to the trend observed in the overall health workforce, women were more heavily represented in entry-level positions in the PHSC workforce than in leadership.

#### Leadership and Decision-Making Norms

Respondents indicated that social and cultural norms may limit women’s career advancement. For example, a female educator from Malawi said that her coworkers believed that to be a leader you had to have an authoritative voice that made students afraid of you. She said her voice was not considered authoritative, so her opportunities to take on leadership roles were negatively impacted. A respondent from the global stakeholder group agreed that women are often not viewed as decision-makers.

Respondents indicated that social and cultural norms may limit women’s career advancement.

*Obviously, you are getting into a male-dominated field and sometimes, you know, when you enter, people don’t really see you as a decision-maker or that you really deserve to be there…maybe they look at you…in the leadership meeting, [and say], “Why don’t you take notes?”* —Female supply chain expert, global level

Female respondents also highlighted a dismissive attitude toward them from their male colleagues. A female PHSC professional from DRC noted that her male colleagues dismissed her before she even had the opportunity to speak.

*At a workshop [in the province]… I was told “oyo aza muasi na biso” (This is our wife). I said that I came not to be someone’s wife, but because I have a job and I have to be given the time to express myself like everyone else.* —Female PHSC professional, DRC

#### Traveling for Training

PHSC roles become more specialized at more senior levels and, therefore, require specialized training. Master’s degrees and specialized certifications are not currently offered at institutions in DRC or Malawi, so individuals must travel internationally to Europe or the United States. Respondents noted that international traveling was more of a barrier for women than men, highlighting that men were considered as fulfilling their role as head of the family, but women were perceived to not love their families. With the lack of equal opportunities for training and advancement due to travel considerations, women may be held back at various points along their career pathways as supply chain professionals and practitioners.

#### Navigating Male-Dominated Spaces

Respondents described when women were able to advance into a management role in PHSC, they experienced additional challenges with supervising their male colleagues, especially in terms of what was considered appropriate interaction. This impacted women’s performance and networking abilities. Male respondents noted that mentoring women made them uncomfortable.

*It becomes challenging to mentor a female if you are a man, because of our culture… because if you spend much time with a woman, you are mentoring her, people think maybe you are not doing work things.* —Male PHSC professional, Malawi

However, respondents noted that when female mentors were available it encouraged more women to enter the PHSC workforce and grow throughout their careers. Respondents noted that engagement and coaching early in a woman’s education was essential to build awareness around the various roles in PHSC and to make informed choices.

## DISCUSSION AND RECOMMENDATIONS

This exploratory research represents the first analysis of the role of gender, specifically in the PHSC workforce, identifying key themes emerging from 2 African countries and triangulating these with perspectives from global stakeholders, including practitioners. The research points to a perceived significant gender imbalance in the PHSC workforce and that this perception plays out across the career pathway, beginning during career preparation, in the hiring and performance of PHSC roles, and in career advancement in the PHSC workforce. Women face barriers related to social and cultural norms on the type of work they are suited for, expectations around domestic duties that may limit their fulfillment of professional duties and opportunities, and their inherent capacity as leaders. They also face practical and environmental challenges in performing their PHSC duties with a risk of harassment with few means of recourse and in navigating male-dominated spaces without strong female role models or networks.

In response to these findings, we recommend that donors, governments, technical partners, academic institutions, and employers take direct action to support women along the PHSC career pathway and continue to explore the impact of gender on preparation, entry, and advancement in the PHSC workforce to further refine evidence-based best practices.

We recommend that donors, governments, technical partners, academic institutions, and employers take direct action to support women along the PHSC career pathway and explore the impact of gender on preparation, entry, and advancement in the PHSC workforce.

### Strengthen Career Pathways

**FIGURE 4:**
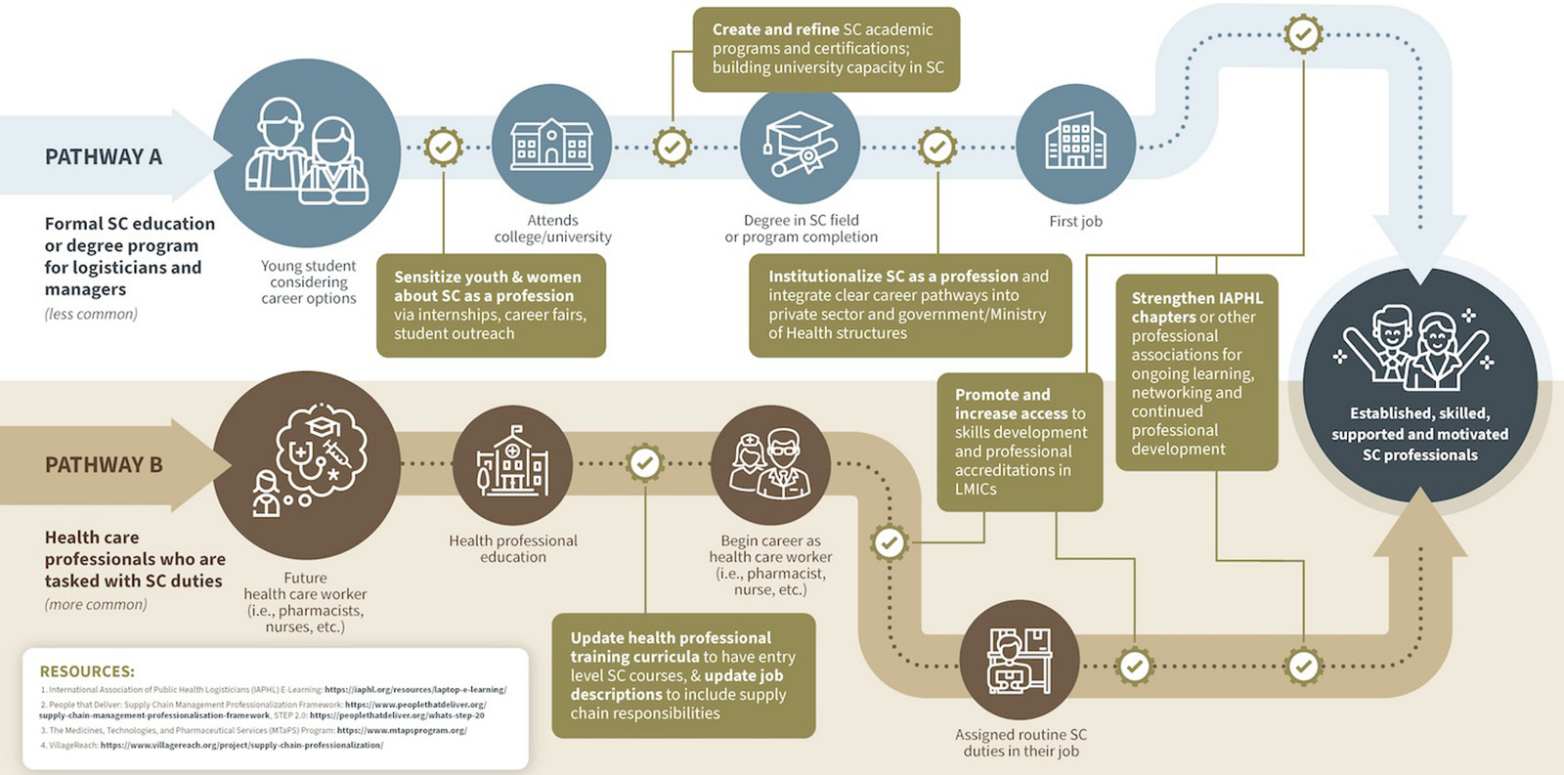
Approaches to Address Barriers Along PHSC Professional Career Pathways Abbreviations: IAPHL, International Association of Public Health Logisticians; LMIC, low- and middle-income country; PHSC, public health supply chain; SC, supply chain.

To equitably establish a pipeline of qualified, motivated PHSC professionals, it is necessary to strengthen career pathways in partnership with ministries of health and ministries of education. WHO indicates that improving access to education for women will enhance their labor participation in the health workforce.^3^ It is necessary to address the key educational findings of this research within the gender context of each country: (1) increase awareness of PHSC professions, (2) address structural and financial barriers, and (3) foster female mentorship and role models. Figure 4 recommends approaches to address barriers faced along the PHSC career pathways illustrated in Figure 1 (Pathway A for supply chain professionals and Pathway B for supply chain practitioners). For Pathway A, we recommend tailored outreach programs for youth and women to understand the roles and responsibilities of a PHSC professional.

Specific recommendations for stakeholders include the following actions.

- Engage women at secondary schools or career fairs by showcasing opportunities available in PHSC and providing information about pathways to enter the industry.
- Plan local supply chain conferences with special sessions for women and include financial or logistical support for event transportation and fees.
- Develop resources (e.g., webinars) and talking points for universities and career counselors dedicated to showcasing the diverse career opportunities within PHSC and female participation in those roles.
- Collaborate with women’s organizations and advocacy groups to promote awareness of careers in PHSC and explore cultural perceptions of these careers among both men and women.
- Introduce PHSC curricula and degree programs at educational institutions and build awareness of these programs among women.
- Create more internship and training opportunities for women in the PHSC workforce, including community health workers, to directly establish a formal career pathway.
- Improve availability of safe and secure housing at educational facilities.
- Increase availability of dedicated scholarships, including provision for supplies such as books, laptops, mobile phones, and phone credit.
- Foster mentorship opportunities (either through an established program or ad hoc) using multiple models (e.g., one-on-one mentoring, peer-to-peer mentoring, or group mentoring).

Although critical to establishing a robust career pipeline for women into PHSC careers, mentorship opportunities are important throughout the career pathway. When women have female mentors, they see what is possible and help to combat gender norms about what women can achieve. At the same time, it is also necessary to consider the time demands placed on professional women, given their household and professional responsibilities and cultural considerations around travel and how women spend their time.

### Improve Recruitment Practices

The research highlighted disparities at the entry point into PHSC careers. To address this, employers must review processes for recruitment to ensure hiring practices are equitable and do not favor men by doing the following.

- Use gender-neutral language in job descriptions and selection criteria.
- Balance gender on interview panels.
- Avoid questions on salary history, gender, and marital status in applications and conduct blind reviews,
- Keep job postings open until a certain number of female candidates have applied, and adjust recruitment strategies or the job posting itself as needed if women are not applying.
- Conduct explicit and implicit bias training for hiring managers.

### Implement Gender-Sensitive Policies

WHO’s Fair Share Report indicates that to increase the value of health and care work for women, stakeholders must focus on gender-equitable investments coupled with gender-transformative policies.^3^ This research identified specific instances related to (1) work-life balance, (2) traveling for work and harassment, and (3) workplace safety that inhibit women’s fulfillment of PHSC roles. The following key policies and practices would help enable more women to work in the PHSC workforce.

- Antidiscrimination or equal opportunity policies that address gender and other forms of discrimination related to recruitment, workplace safety, and pay.
- Gender-responsive leave policies that offer maternal, paternal, and family leave options.
- Sexual harassment policies that protect women and create reporting mechanisms when they face harassment in educational or work settings. Countries must adopt zero-tolerance policies around harassment and discrimination and provide mechanisms to address these issues across the entire career pathway—from education through advancement.
- A list of safe accommodation facilities with clean and accessible washrooms for work-related travel.
- Policies to ensure that women are accompanied by female colleagues for travel to improve security during work-related travel.

### Elevate Female Decision-Making

Once women begin working in the PHSC workforce, their advancement opportunities may be limited due to cultural norms and the need to navigate male-dominated spaces. Leaders in the PHSC workforce need to address barriers to entry and purposefully select and include women in their decision-making bodies, technical working groups, and committees. Ensuring gender-equitable selection of participants for capacity-building opportunities (e.g., training, site visits, and conference participation) can get more women on the path toward decision-making roles.

### Collect and Use Gender-Disaggregated Data

Improving the gender imbalance in the PHSC workforce requires better data to track improvements and changes to the workforce over time, including understanding gender balance at all levels of the PHSC workforce. The data could be collected from a human resources information system, such as the iHRIS health workforce registry, or integrated into other routine data collection efforts. These systems can be leveraged to collect gender- and age-disaggregated data to identify areas with significant imbalances across the PHSC career pathway. Supply chain training organizers should track participation by sex and age at multiple levels of the workforce to inform participant selection for future training or identify the need for strategies to build capacity for women outside formal training.

Donors can help ensure that gender-disaggregated PHSC data are collected by partners and the Ministry of Health by requiring it for reporting. Educational institutions should track the gender breakdown of students enrolled in PHSC programs, course performance, graduation rates, and, ideally, employment rates after graduation.

### Limitations

As an exploratory study using primarily qualitative methods, the results presented are the perceptions of the survey respondents and key informant interviews. The results are not fully representative of either country or of other country contexts. The researchers used themes to develop preliminary recommendations to promote action, but these recommendations need to be validated by governments, other practitioners, and PHSC professionals themselves. These recommendations will need to be refined and validated to reflect emerging best practices in this nascent field.

## CONCLUSION

This exploratory research demonstrates that a perceived gender imbalance exists in the PHSC workforce, beginning during career preparation and extending to career entry, retention, and advancement. Gender balance in the PHSC workforce contributes to building high-performing supply chains that are equitable, people-centered, resilient, and sustainable. Supply chains with these attributes are a critical component of responsive primary health care systems that meet the needs of the most under-reached communities—particularly among women, adolescents, and children—where global health outcomes remain the lowest.

Many opportunities exist for governments, nongovernmental organizations, funders, and others in the supply chain and human resources for health communities to address the gender imbalance in the PHSC workforce at the global and country levels. Governments can address policy issues that keep women out of the PHSC workforce, and employers can actively engage women during the early education stage and create mentorship programs for women in partnership with both the ministries of health and education. Academic institutions can intentionally recruit female students and make sure they have access to housing, scholarships, and safe environments. Implementing partners and donors can assist the government in establishing tailored programs to recruit and support women along the PHSC career pathway. Through active engagement and intentional considerations of gender, all stakeholders can play a role in encouraging more women can participate in the PHSC workforce.

## Notes

**Peer Reviewed**
